# Expert consensus on robotic surgery for colorectal cancer (2015 edition)

**DOI:** 10.1186/s40880-016-0085-3

**Published:** 2016-02-25

**Authors:** Jianmin Xu, Xinyu Qin

**Affiliations:** Department of General surgery, Zhongshan Hospital, Fudan University, Shanghai, P. R. China

Professional Committee of Colorectal Surgeons, Chinese College of Surgeons of Chinese Medical Doctor Association

Robotic and Laparoscopic Surgery Committee of Chinese Research Hospital Association

Editorial board members of “Expert consensus on robotic surgery for colorectal cancer (2015 edition)”

Group leaders: Jianmin Xu, Xinyu Qin, Peiwu Yu

Assistants: Guodong He (angelhgd@163.com), Qingyang Feng (fqy198921@163.com)

Members (in alphabetic order by pinyin of last name):

**Table:** 

Longwei Cheng	chenglongwei0509@163.com	Jilin Cancer Hospital
Xiaohui Du	duxiaohui301@sina.com	The General Hospital of the People’s Liberation Army
Wenxian Guan	15850502391@163.com	Nanjing Drum Tower Hospital
Yulong He	Ylh@medmail.com.cn	The First Affiliated Hospital of Sun Yat-sen University
Baoqing Jia	baoqingjia@126.com	The General Hospital of the People’s Liberation Army
Kewei Jiang	jiangkewei@pkuph.edu.cn	Peking University People’s Hospital
Zhiwei Jiang	surgery34@163.com	Nanjing General Hospital of Nanjing Military Command
Taiyuan Li	jylitaiyuan@sina.com	The First Affiliated Hospital of Nanchang University
Yisheng Pan	yisheng.pan@pkufh.cn	Peking University First Hospital
Xinyu Qin	qin.xinyu@zs-hospital.sh.cn	Zhongshan Hospital of Fudan University
Huizhong Qiu	qiuhzpumch@163.com	Peking Union Medical College Hospital
Yaqing Si	siyaqing2008@163.com	The First Affiliated Hospital of Zhengzhou University
Bo Tang	tangbo@sina.cn	Southwest Hospital
Shiliang Tu	tushiliang@126.com	Zhejiang Provincial People’s Hospital
Kang Wang	cyh633@163.com	Sichuan Provincial People’s Hospital
Xin Wang	wangxin_guo@hotmail.com	Peking University First Hospital
Ziqiang Wang	wangzqzyh@163.com	West China Hospital, Sichuan University
Ye Wei	13818661815@126.com	Zhongshan Hospital of Fudan University
Guosheng Wu	guosheng_w@yahoo.com	Xijing Hospital
Jianmin Xu	xujmin@aliyun.com	Zhongshan Hospital of Fudan University
Yingjiang Ye	yeyingjiang@pkuph.edu.cn	Peking University People’s Hospital
Peiwu Yu	Yupeiwu01@sina.com	Southwest Hospital
Weitang Yuan	13673384555@163.com	The First Affiliated Hospital of Zhengzhou University
Dongzhu Zeng	zdz1140@163.com	Southwest Hospital
Wei Zhang	Weizhang2000cn@163.com	Changhai Hospital
Xiaoqiao Zhang	xqz@vip.163.com	Jinan Military General Hospital
Xuefeng Zhang	zxfpwk@163.com	The General Hospital of Shenyang Military
Min Zhong	Drzhongming@hotmail.com	Renji Hospital Shanghai Jiaotong University School of Medicine
Yanbing Zhou	zhouyanbing999@aliyun.com	The Affiliated Hospital of Qingdao University

The Chinese version of “Expert consensus on robotic surgery for colorectal cancer (2015 edition)” has been published on the *Chinese Journal of Digestive Surgery* (2015, Volume 14, Issue 11) and the *Chinese Journal of Practical Surgery* (2015, Volume 35, Issue 12). The English version of this consensus is published on the *Chinese Journal of Cancer* with permission from the above two Chinese journals.

The robot-assisted colorectal surgery in China is still at the initial stage. The “Expert consensus on robotic surgery for colorectal cancer” was written to provide a guideline for surgeons to perform this surgery.

## Features and advantages of a robot-assisted surgical system

### Technical features

Three integrated components compose the robot-assisted surgical system: a video tower, a patient cart with robotic arms, and a surgeon console. The video tower displays high-definition 3-dimensional vision for a true perception of depth during surgery, increasing the surgeon’s confidence as a result of the superior view of the tissue plains and the critical anatomy. The patient cart is composed of multiple components, including one camera arm and three robotic arms. The robotic arms are designed with unique wristed architecture that provides 540 degrees of manipulation, a range of motion greater than even the human wrist. The system enables the surgeon to perform with dexterity and with very deliberate motion control of the instruments to pursue precise surgical tasks. Sitting at the surgeon console, the surgeon can control the movement of the patient cart precisely and seamlessly and also avoid standing for long periods during surgery, reducing physiological fatigue [1–3]. Moreover, the master controllers provide tremor filtration to stabilize the surgical procedure.

Despite the above advantages of robotic surgery today, there are still areas to be improved, such as reducing the time to connect all system cables, exchanging the robotic and camera arms with each other, reducing the size of the arms and expanding their range, strengthening the feedback mechanism during surgery, and reducing material and maintenance costs.

### Clinical application

Robotic technology has been maturely applied to rectum and sigmoid colon resection. A large number of retrospective studies and meta-analyses as well as some randomly controlled trials with small sample sizes showed the following advantages of robotic surgery: the accurate operation and precise separation of the rectum from its surrounding tissues; the wristed architecture can overcome the problems caused by the relatively blind angle when surgeons dissociate the lateral spatium in the lower rectum using straight bar instruments, ensuring the total resection of the mesorectum; rapid recovery of gastrointestinal function after surgery; better protection of the pelvic autonomic nerve for urinary and sexual functions; less estimated intraoperative blood loss; and lower conversion rate to open surgery and similar postoperative complication incidence and hospital stays as compared with laparoscopic surgery [4–11].

Compared with laparoscopic and open surgery, robotic surgery demonstrated comparable oncological parameters, such as lymph node detection rate, distal mesorectal margin positive rate, local recurrence rate, and long-term survival rate, and it possessed potential superiority in reducing the positive rate of the circumferential resection margin [5, 7].

Robot-assisted right hemicolectomy is still in its developing stage. A retrospective study and meta-analysis showed that, compared with laparoscopic surgery, robot-assisted right hemicolectomy benefited patients with better postoperative recovery and less blood loss and demonstrated similar conversion rate to open surgery, complication incidence, and hospital stays [12, 13]. In terms of oncologic parameters, lymph node detection rate and positive surgical margin rate in the robotic surgery group were similar to those in the laparoscopic surgery group. To date, long-term survival outcomes after robot-assisted right hemicolectomy have not been reported yet. Robotic surgery for colon cancer in other segments (left half of the transverse colon, left colic flexure, and descending colon) were rarely reported, and the surgery’s advantages need further evaluation.

## Indications and contraindications for robot-assisted colorectal surgery

The indications for this surgery are similar to those for conventional laparoscopic surgery. The contraindications are as follows:

1. General anesthesia intolerance, e.g., patients with severely insufficient heart, lung, or liver function;
2. Severe coagulation disorder;
3. Pregnancy;
4. Extensive abdominal or pelvic metastasis that is difficult to dissect with a robotic system;
5. Tumor obstruction with obvious distention;
6. Tumor perforation with acute peritonitis;
7. Difficult to puncture due to extensive abdominal adhesion;
8. Moribund condition, massive ascites, intra-abdominal hemorrhage, or shock; and
9. Severe obesity, with a body mass index (BMI) >40 kg/m^2^ (extended puncture device and surgical instruments in the robotic surgical system are unavailable).

## Perioperative preparation

### Patients

Patient preparation includes bowel preparation and prophylactic administration of antibiotics during anesthesia induction. General anesthesia with endotracheal intubation is adopted during the operation, and a urethral catheter is indwelled; a nasogastric tube can be placed as well when necessary. Other preoperative preparations are similar to those for conventional surgery.

### Instruments

1. The robotic arms interface with its specifically designed supporting components, and the laparoscopic instruments can also be used by assistants in surgery.
2. The robotic arms can selectively hold different instruments: hot shears (monopolar curved scissors), electrocautery, harmonic scalpel, fenestrated grasper, fenestrated bipolar forceps, Maryland bipolar forceps, grasping retractor, and so on.
3. Laparoscopic instruments used by the assistant include laparoscopic bowel forceps, scissors, suction irrigation sets, 5 mm Ligasure V, Hemo-lock clip applier, and linear cut stapler.
4. The instruments for extracorporeal anastomosis are the surgical incision protector and circular stapler.
5. Sterile drapes for robotic arms.

### Robotic system

1. Conduct a system power-on self-test.
2. Ensure that all robotic instruments are present and the system is in good conditions. In particular, check if the arm motion is flexible, the wrist and instrument movement is not restricted, and the scissors and forceps are normal.
3. Install the sterile drapes for the robotic system.
4. Once the light from the illuminator is delivered to the endoscope, set the white balance, adjust the focus, and calibrate the camera. After that, heat the endoscope (not beyond 55 °C) to avoid fogging.
5. Arrange equipment around and above the operating table and properly fix equipment power transmission lines to avoid affecting the motion of the robotic arms.
6. If the robotic arms collide during the procedure, reposition them.
7. The surgeon can adjust the height and tilt of the stereo viewer and move the armrest up and down by controlling the console screen.

## Surgical procedures for robot-assisted colorectal cancer resections

### Robot-assisted radical resection of rectal and sigmoid cancers

Robotic surgical procedures are used for rectal and sigmoid cancers, including radical resection of sigmoid cancer, (low) anterior resection and abdominoperineal resection of rectal cancer.

#### Surgical position

The herringbone position or the modified lithotomy position [14] is used for radical resection of sigmoid cancer and (low) anterior resection of rectal cancer; the lithotomy position is used for abdominoperineal resection of rectal cancer. After the patient is secured, the operating table is turned to the Trendelenburg position with the right side inclined downward. The patient’s left leg is placed downward to avoid colliding with the robotic arms.

#### Trocar number and location

**Fig. 1:**
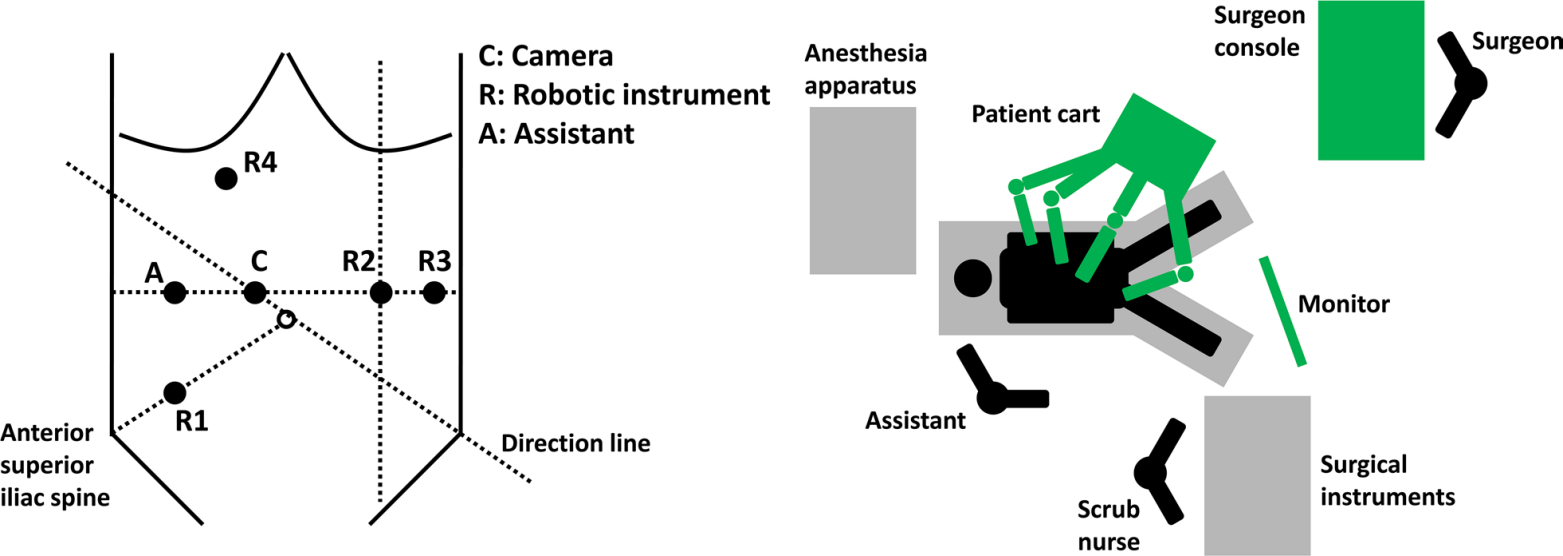
Trocar location and operating room setup for robot-assisted radical resection of rectal or sigmoid cancer

Usually, 4–5 trocars are placed for the surgery: 1 for the camera (Trocar C), 3 for the robotic arms (Trocar R1, R2, and R3), and 1 for the assistant (Trocar A). If the left colic flexure is mobilized during the surgery, Trocar R4 should be used instead of Trocar R2 for the robotic arms. Details are shown in Fig. 1.

1. Trocar C: 12 mm in diameter, placed 3–4 cm to the upper right of the umbilicus.
2. Trocar R1: 8 mm in diameter, placed at the McBurney’s point (one-third of the distance from the right anterior superior iliac spine to the umbilicus).
3. Trocar R2: 8 mm in diameter, placed at the intersection of the left mid-clavicular line and the horizontal line through Trocar C.
4. Trocar R3: 8 mm in diameter, placed at the intersection of the left anterior axillary line and the horizontal line through Trocar C. This trocar is always used to help mobilize the lower rectum.
5. Trocar R4: 8 mm in diameter, placed 3–4 cm below the xiphoid process, in the middle of the anterior midline and the right mid-clavicular line. This trocar is used to mobilize the left colic flexure.
6. Trocar A: 5 or 12 mm in diameter, placed at the intersection of the vertical line through the McBurney’s point and the horizontal line through Trocar C.

The location of Trocar C is relatively fixed. The locations of other trocars could be adjusted according to the tumor site, the patient’s body shape, and the surgeon’s operating habits, although the operating center should be fixed to the tumor. The adjacent trocars should be 8–10 cm from each other to avoid collisions of robotic arms. All measurements should be based on the tension after the pneumoperitoneum. Trocars R1, R2, and/or R3 are used to mobilize the rectum, and trocars R1, R4, and/or R3 are used to mobilize the left colic flexure.

#### Abdominal exploration

After establishing pneumoperitoneum at a pressure of 8–15 mmHg, the camera on either the laparoscope or the surgical robot can be used for abdominal exploration. If tissue adhesions are found to interfere with the trocar puncture, laparoscopic instruments should be used to release them. Before the robot system is connected, the patient’s position should be adjusted to ensure sufficient exposure of the operative field.

#### Robot system connections

The patient cart is placed on the left side of the patient, with the direction line through the left anterior superior iliac spine, trocar C, and the center column of the patient cart (Fig. 1). All robotic arms should surround the operating center: the camera arm is located in the middle, and the instrument arms on the sides, with joints fully extended outward to avoid collisions. The digital pattern on the instrument arms should face straight ahead. When connecting robotic arms with trocars, movements should be gentle to avoid pulling up the trocars. After the robotic arms are fixed, neither the patient nor the operating table should be moved again.

#### Surgical procedure

##### 1. Exposure of the operative field

The medial-to-lateral approach is recommended for the surgery. To improve the exposure of operative field, the uterus could be suspended in female patients, and the bladder could be suspended in male patients. With Trocar A, the assistant moves the small intestine and greater omentum to the right upper abdominal cavity. The mesenteric junction of the rectosigmoid and posterior peritoneum is tilted upward and outward to identify the abdominal aortic bifurcation.

##### 2. Division of vessels

A “mesenteric window” is opened just at the sacral promontory plane. The inferior mesenteric vessels are dissected through the space between the visceral and parietal peritoneum (the Toldt’s space) and ligated at their origin points using Hemo-locks. Lymph nodes are also swept clearly.

##### 3. Mobilization of the side peritoneum

The sigmoid is tilted rightward, and the Toldt’s space is dissected. The left ureter should be exposed and safeguarded during the mobilization.

##### 4. Mobilization of the left colic flexure

**Fig. 2:**
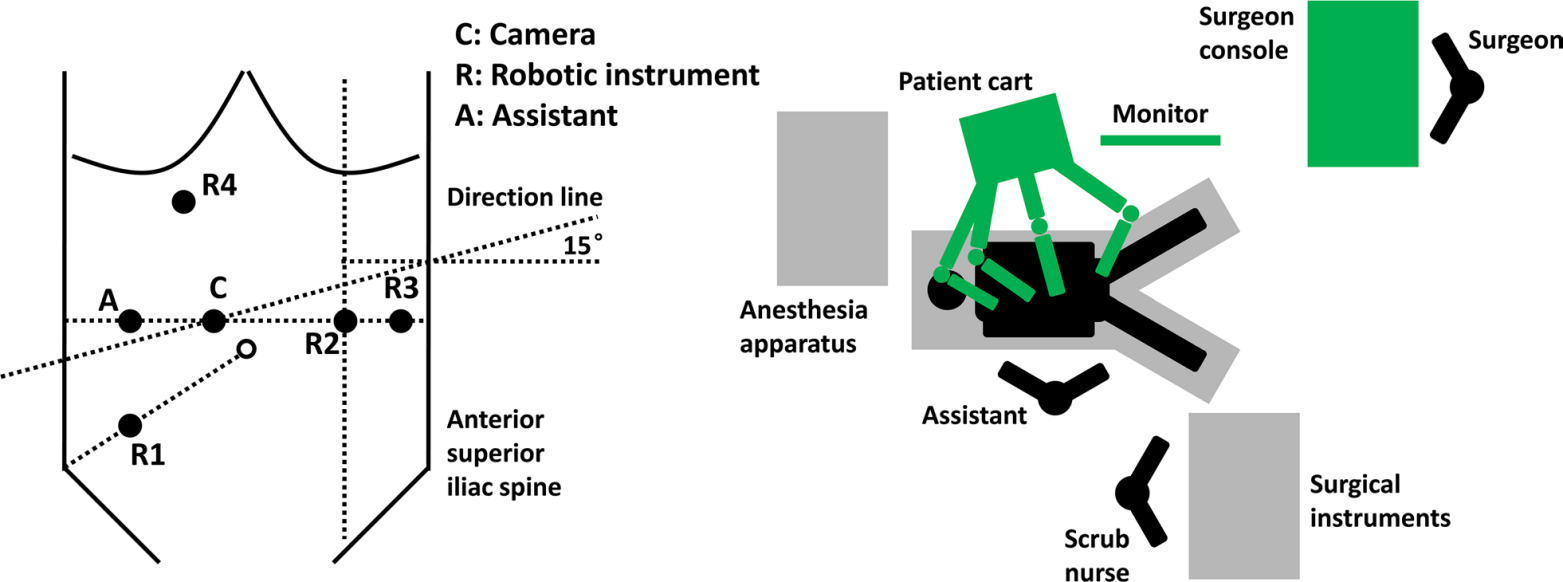
Trocar location and operating room setup for left colic flexure mobilization in robot-assisted radical resection of rectal or sigmoid cancer

First, the robotic arms should be removed. Then, the patient cart should be replaced beside the left shoulder of the patient, with the direction line through Trocar C and at an angle of 15° from the horizontal line (Fig. 2). The surgical robot system should also be re-connected. Trocars R1 and R4 are used to mobilize the left colic flexure. For patients with short sigmoid as confirmed in preoperative evaluation, the left colic flexure can be mobilized before the rectosigmoid.

##### 5. Mobilization of the descending and sigmoid colon

The descending and sigmoid colon are mobilized along the prerenal fascia on the surface of the ureter. The nerve plexus should be safeguarded during the mobilization. The mesocolon is cut according to the proximal resection margin.

##### 6. Mobilization of the rectum

The rectum is mobilized in a circular route, following the principles of total mesorectal excision (TME). The mobilization starts from the posterior rectum wall and gradually extends to the lateral sides; the anterior rectum wall is dissected last. For patients with contracted pelvis, lateral sides can also be dissected after the posterior and anterior wall. Trocar R3 is always used to help tilt the rectum. The tension of the arms should be controlled to avoid soft tissue avulsion. The tumor site is the basis for determining whether to open the peritoneal reflection and the length of the mobilized rectum, and the rectum can be mobilized till the levator plane if necessary. In mobilizing the lower rectum, electric scissors and hook may be more flexible.

##### 7. Division of the distal mural margin

The distal mural margin can be dissected using electric scissors and hook or ultrasonic energy instruments. The margin should be more than 2 cm below the inferior edge of the tumor.

##### 8. Anastomosis

Extracorporeal or intracorporeal anastomosis should be selected according to the tumor site and the patient’s body shape. In extracorporeal anastomosis, the incision is made in the left lower abdomen. The bowel with the tumor is pulled out for anastomosis under direct vision. A reinforcement suture can be made if necessary. In intracorporeal anastomosis, the tumor is removed through a small incision in the left lower abdomen or an enlarged puncture incision. A purse-string suture is placed in the proximal resection margin, and the anvil is tied around the margin of the colon. Then, the proximal colon along with the anvil is returned to the abdomen. The incision is closed, and the pneumoperitoneum is reestablished. The circular stapler is inserted through the anus, and the anastomosis is made under visualization of the surgical robot system. For small tumors, the affected bowel can be pulled out through the anus to remove the tumor. The anvil is tied to the proximal resection margin and is returned through the anus. The anastomosis is made under visualization of the surgical robot system and is checked for any leaks by air or methylene blue perfusion. A reinforcement suture can be made under visualization of the surgical robot system if necessary.

##### 9. Perineal surgery and colostomy

For patients who are undergoing abdominoperineal resection, perineal surgery is continued manually after the rectum is mobilized till the levator plane. The procedure is the same as that for conventional open surgery. The affected bowel is removed from the perineal incision, and the robotic arms are also removed. The colostomy is then performed manually. The perineal incision is closed after the perineal surgery colostomy is completed.

##### 10. Incision closure

To close the pelvic peritoneum, the pneumoperitoneum should be reestablished, and the surgical robot system should also be reconnected. The abdominal cavity is irrigated with normal saline or distilled water and drain adequately. Then, all incisions are closed.

### Robot-assisted radical resection of left-sided colon cancer

Robotic surgical procedures are used for cancers located at the left transverse colon, left colic flexure, and descending colon.

#### Surgical position

The herringbone position or the modified lithotomy position [14] is used for the surgery. After the patient is secured, the operating table is turned to the reverse Trendelenburg position with the right side inclined downward. The patient’s left leg is placed downward to avoid collision with the robotic arms.

#### Trocar number and location

**Fig. 3:**
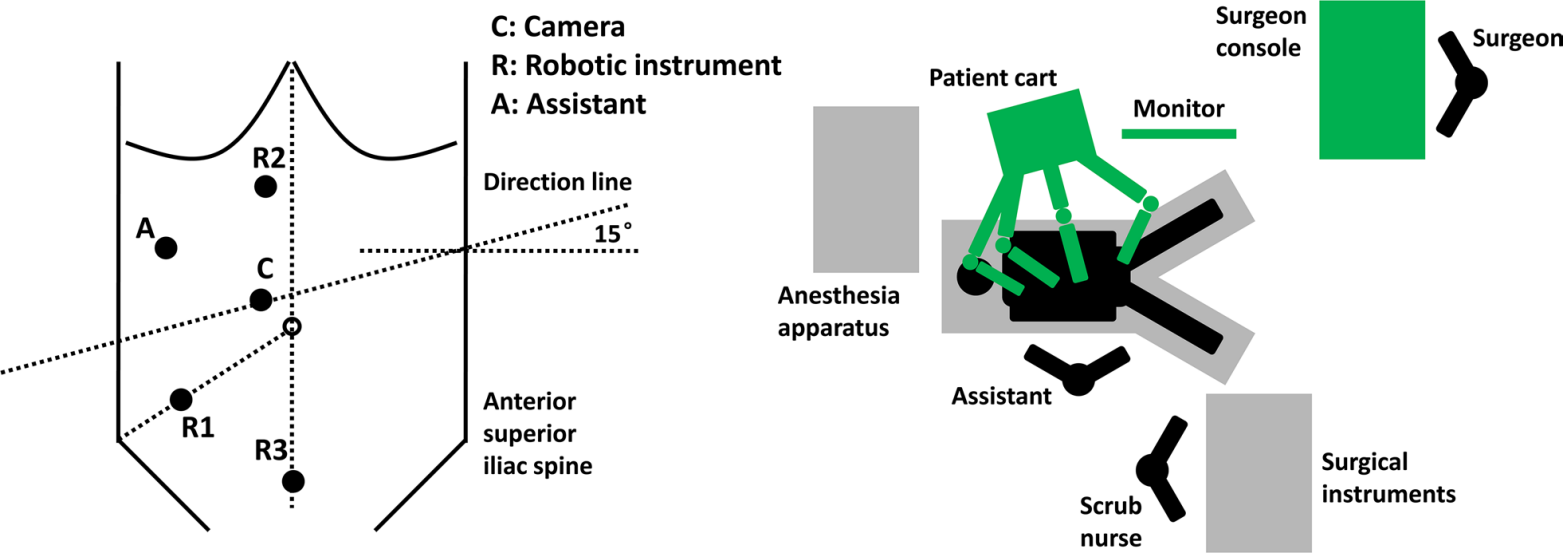
Trocar location and operating room setup for robot-assisted radical resection of left-sided colon cancer

Usually, 5 trocars are placed for the surgery: 1 for the camera (Trocar C), 3 for the robotic arms (Trocars R1, R2, and R3), and 1 for the assistant (Trocar A). Details are shown on Fig. 3.

1. Trocar C: 12 mm in diameter, placed 3–4 cm to the upper right of the umbilicus.
2. Trocar R1: 8 mm in diameter, placed at the McBurney’s point (one-third of the distance from the right anterior superior iliac spine to the umbilicus).
3. Trocar R2: 8 mm in diameter, placed at the right side of the anterior midline, 3–4 cm below the xiphoid process. Ensure that it is placed above the transverse colon.
4. Trocar R3: 8 mm in diameter, placed on the anterior midline, 3–4 cm above the symphysis pubis.
5. Trocar A: 5 or 12 mm in diameter, placed outside the right midclavicular line in the middle of Trocar C and Trocar R2.

The location of Trocar C is relatively fixed; the locations of other trocars could be adjusted according to the tumor site, the patient’s body shape, and the surgeon’s operating habits. The operating center should be fixed to the tumor. The adjacent trocars should be 8–10 cm from each other to avoid collisions of the robotic arms. All measurements should be based on the tension after the pneumoperitoneum.

#### Abdominal exploration

The same procedures apply as those mentioned above in “Robot-assisted radical resection of rectal and sigmoid cancers” section.

#### Robot system connections

The patient cart is placed beside the left shoulder of the patient, with the direction line through Trocar C and the center column of the cart at an angle of 15° from the horizontal line (Fig. 3). Other considerations are the same as those mentioned above in “Robot-assisted radical resection of rectal and sigmoid cancers” section.

#### Surgical procedure

##### 1. Exposure of the operative field

The medial-to-lateral approach is recommended for the surgery. Through Trocar A, an assistant moves the small intestine and greater omentum to the right abdominal cavity. The mesenteric junction of the descending and sigmoid colon is tilted upward and outward, and the junction of the sigmoid colon and rectum is tilted downward and outward to identify the abdominal aortic bifurcation.

##### 2. Division of vessels

A “mesenteric window” is opened just at the sacral promontory plane. The first and second branches of the sigmoid vessels and the left colic vessels are dissected through the Toldt’s space along the inferior mesenteric vessels. The vessels are ligated at their origin points from the inferior mesenteric vessels, using Hemo-locks. Lymph nodes are also swept clearly.

##### 3. Mobilization of the descending colon

From the left side of the inferior mesenteric vein, the descending colon is mobilized through the Toldt’s space between the mesocolon and the left prerenal fascia. Mobilization is from up to down, or from up to down and from the inside to the outside, on the surface of the left spermatic or ovarian vessels and the left ureter.

##### 4. Mobilization of the left colic flexure

The left colic flexure is mobilized through the Toldt’s space inward and upward. The left branch of middle colic artery is ligated, and the left gastrocolic and splenocolic ligaments are dissected to fully mobilize the left colic flexure.

##### 5. Mobilization of the sigmoid colon and upper rectum

The descending and sigmoid colon are fully mobilized through the Toldt’s space; the upper rectum can also be mobilized if necessary. The length of resected bowel is decided, and the affected bowel is dissected.

##### 6. Anastomosis

The affected bowel is pulled out through a left rectus incision to remove the tumor. An alternative is side-to-side or end-to-side anastomosis of the transverse and sigmoid colon.

##### 7. Incision closure

The abdominal cavity is irrigated with normal saline or distilled water and drain adequately. Then, all incisions are closed.

### Robot-assisted radical resection of right-sided colon cancer

Robotic surgical procedures are used for cancers located at the cecum, ascending colon, hepatic flexure, and right-sided transverse colon.

#### Surgical position

Supine position is used for radical resection. The patient should be set close to the cranial side of the operating table, and the anterior superior spine should be higher than the middle plane. After the patient is secured, the operating table is turned to the Trendelenburg position with an angle of 15°–30°, and left side downward with an angle of 10°–15°.

#### Trocar number and location

**Fig. 4:**
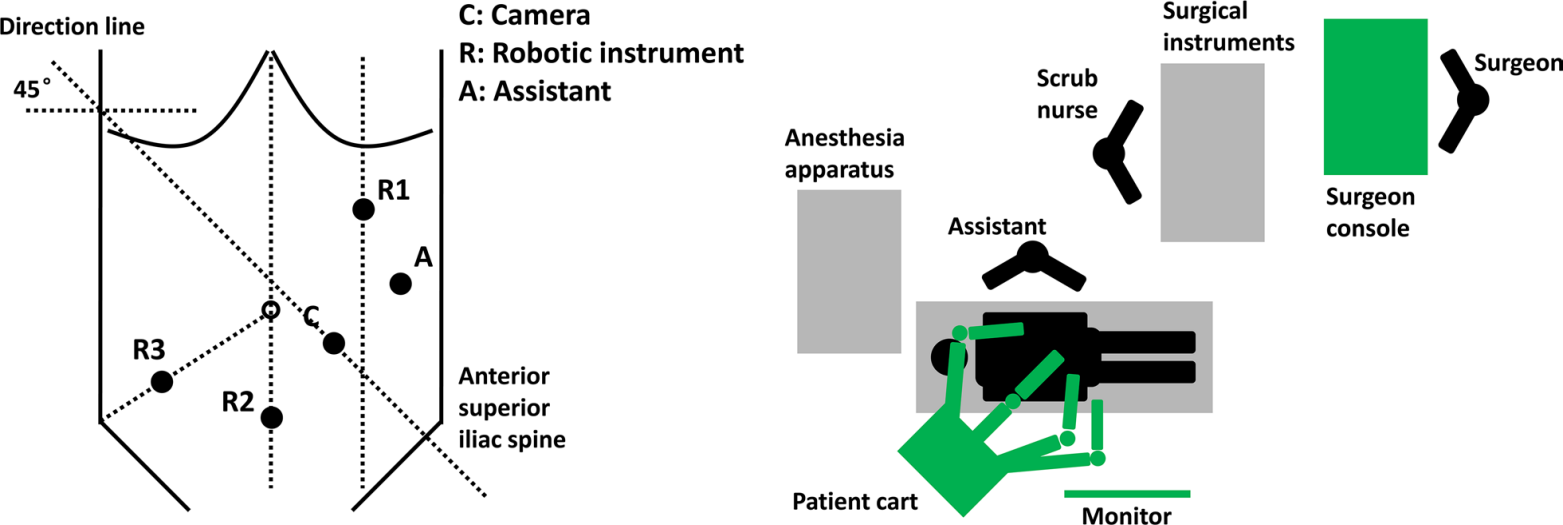
Trocar location and operating room setup for robot-assisted radical resection of right-sided colon cancer

Usually, 5 trocars are placed in the surgery: 1 for the camera (Trocar C), 3 for the robotic arms (Trocar R1, R2, and R3), and 1 for the assistant (Trocar A). Details are shown on Fig. 4.

1. Trocar C: 12 mm in diameter, placed 3–4 cm to the lower left of the umbilicus.
2. Trocar R1: 8 mm in diameter, placed on the left midclavicular line, 7–8 cm below the costal margin.
3. Trocar R2: 8 mm in diameter, placed on the anterior midline, 6–8 cm above the symphysis pubis.
4. Trocar R3: 8 mm in diameter, placed at the McBurney’s point (one-third of the distance from the right anterior superior iliac spine to the umbilicus).
5. Trocar A: 5 or 12 mm in diameter, placed outside the left midclavicular line, 6–8 cm below Trocar R1, and more than 8 cm away from Trocar C.

The location of Trocar C is relatively fixed. The locations of other trocars could be adjusted according to the tumor site, the patient’s body shape, and the surgeon’s operating habits. The operating center should be fixed to the tumor. The adjacent trocars should be 8–10 cm away from each other, avoiding collisions of robotic arms. All measurement should be based on the tension after pneumoperitoneum.

#### Abdominal exploration

The same as mentioned above in “Robot-assisted radical resection of rectal and sigmoid cancers” section.

#### Robot system connections

The patient cart is placed beside the right shoulder of the patient, with the direction line through Trocar C and the center column of the patient cart, with an angle of 45° from the horizontal line (Fig. 4). There should be enough space beside the patient’s hip to avoid collision with robotic arms when mobilizing the hepatic flexure. Other considerations are the same as those mentioned above in “Robot-assisted radical resection of rectal and sigmoid cancers” section.

#### Surgical procedure

##### 1. Exposure of the operative field

The medial-to-lateral approach is recommended for the surgery. With Trocar A, the assistant moves the small intestine to the left abdomen, and lift the right mesocolon to expose the junction of the ileocolic artery and the superior mesenteric vein.

##### 2. Division of vessels

Dissection is performed upward along the superior mesenteric vessels to divide each branch and sweep the lymph nodes. Hemo-locks are used to ligate the ileocolic vessels, right colic vessels, and (the right branch of) middle colic vessels. For tumors located at or near the hepatic flexure which need expanded surgery, the right gastroepiploic vessels are also ligated at the inferior edge of pancreas.

##### 3. Mobilization of the ascending colon

From the right side of the superior mesenteric vein, the ascending colon is mobilized through the Toldt’s space between the mesocolon and right prerenal fascia. Mobilization is performed from downside to upside, from inner to outside, on the surface of the right spermatic or ovarian vessels, right ureter pancreas, and duodenum.

##### 4. Mobilization of the hepatic flexure

Gastrocolic ligament is opened to mobilize the hepatic flexure rightward. The right gastroepiploic vessels and corresponding lymph nodes should be swept if the tumor locates at or near the hepatic flexure. More than 10 cm length of greater omentum should be dissected and cut off.

##### 5. Mobilization of the side peritoneum

From the ileocecal junction, the right-sided peritoneum is mobilized upward and converged with the hepatic flexure.

##### 6. Anastomosis

The mesentery of the colon and small intestine is mobilized till resection margin. The bowel is resected according to the tumor site. Intracorporeal anastomosis and extracorporeal anastomosis with assistant incision are both feasible. In intracorporeal anastomosis, the terminal ileum is get close to the colon. Linear stapler is used for a side-to-side anastomosis. Then another linear stapler is used to cut off the specimen.

The affected bowel is pulled out through the left rectus incision to remove the tumor. It is alternative to make side-to-side or end-to-side anastomosis of the transverse and sigmoid colon. Circular stapler can also be used for end-to-side anastomosis.

##### 7. Incision closure

The abdominal cavity is irrigated with normal saline or distilled water and put drainage adequately. Then, all incisions are closed.

## Robotic multiple organ resection

Local invasion and distant metastasis are common in patients with colorectal cancer, and thus, multiple organ resection is an important measure for radical resection of colorectal cancer. Robotic surgery is also applicable in combination resection [15], although it should only be performed by experienced surgeons after a multidisciplinary team consultation. For locally advanced colorectal cancer with invasion of adjacent organs (mainly rectal tumors invading the urinary bladder, ovary, and uterus), robotic surgery can be performed to resect the organs without withdrawing and re-fixing the robotic arms. This type of surgery can also be applied in synchronous resection of colorectal cancer with distant metastases such as liver or lung metastases that need re-punching and re-docking after one lesion being resected. Additionally, during the resections of different lesions, the same ports should be used when possible to minimize trauma. Today, robotic liver resection has been demonstrated to be safe and effective [16, 17], but the long-term effects of synchronous resection of colorectal cancer and liver metastasis lesions remain to be evaluated.

## Prevention and treatment of complications

Complications of robot-assisted colorectal surgery are similar to those of conventional laparoscopic surgery, but there are also unique robotic surgery complications.

### Intraoperative complications

#### Puncture injury

Vascular and bowel injury.

Prevention: Pay attention to puncturing; the open access technique is recommended.

Treatment: Once bowel injury occurs, convert to open surgery and repair the injured intestine immediately.

#### Complications associated with the pneumoperitoneum

Cardiopulmonary dysfunction and hypercapnia.

Prevention: Close monitoring during operation is needed to avoid extensive subcutaneous emphysema. In addition, it is also necessary to maintain muscle relaxation and shorten the operation time.

Treatment: Finish the operation as soon as possible, and exhaust CO_2_ gas in the abdominal cavity.

#### Intraoperative bleeding related to vascular injury

Injury to the superior and inferior mesenteric vessels or their branches, the anterior sacral vein, the pelvic vessels, and so on.

Prevention: Have a good grasp of the normal anatomy and anatomic variances, pay special attention to surgical anatomic planes and vascular anatomy, and skillfully use the energy platform.

Treatment: Stay calm during the operation, cooperate closely with the team, and properly use the hemostatic tools.

#### Injury to adjacent organs

Injury to the ureter, bladder, prostate, urethra, vagina, duodenum, liver, spleen, gallbladder, and so on.

Prevention: Learn about the normal anatomic structure and dissect along the accurate surgical planes to avoid injury.

Treatment: Timely detection and treatment.

#### Complications related to intestinal anastomosis and enterostomy

Injury to the intestine during exposure, intestinal rupture, dehiscent and bleeding anastomosis, and bleeding stoma.

Prevention: Pay attention to operative skills, including appropriate separation and excision. Choose proper linear cutting and circular stapling devices.

Treatment: Suture and repair the injured area. Use a proper stapler and keep the operation tips in mind. Reinforce the sewing of stoma, prophylactic enterostomy, decompressive tube drainage, and so on.

#### Failure or inflexibility of the arms

A possible reason is the lack of a perfect fit when the instruments were installed or exchanged.

Solution: Reinstall or exchange the instruments.

#### Tissues embedded into the conjunction of operative devices

The multi-angle movement of the robotic arms may cause clipping of the tissues at the conjunctions.

Solutions: Avoid normal neighboring tissues; resect the tissues if appropriate; repair the injured intestinal wall.

#### Rupture of hot shear holster

The rupture of the holster can cause accident burns at the rupture site.

Solutions: Periodically change the holster; check the holster during the operation when a burn occurs, and replace the damaged one immediately.

#### Target anatomy is unreachable

We may find that the devices cannot reach the target anatomy during an operation.

Solutions: Check whether any contacts or collisions are affecting the movement of the robotic arms and whether the external length of the cannula is too long to allow the movement of the robotic arms.

### Postoperative complications

#### Anastomotic fistula

Anastomotic fistula happens most frequently after low and ultra-low rectal anterior resection.

Prevention: Prophylactic enterostomy, close the pelvic peritoneum, place an anal tube for drainage, and so on.

Treatment: If peritonitis is local, keep drainage unobstructed and use systemic antibiotics. Surgical exploration, abdominal lavage and drainage combined with enterostomy are recommended once acute diffuse peritonitis has occurred.

#### Intestinal obstruction

This can happen at any time after the operation and in any part of the bowel. The frequency of intestinal obstruction in the early stage after operation is reduced by robotic surgery as compared with open surgery.

Prevention: Close the mesentery as far as possible and avoid incomplete closure; promote early postoperative ambulation.

Treatment: Implement surgical exploration after the intestinal obstruction is diagnosed and when there is no remission with conservative treatment.

#### Dysfunction of urination and sex

Prevention: The key point is the intended exposure and protection of the pelvic nerve during the operation.

#### Hernia of port-site and stoma hernia

This often occurs in incisions with a diameter more than 10 mm, especially in the elderly with weak abdominal walls.

Prevention: Suture the incisions longer than 10 mm and avoid increasing intra-abdominal pressure.

Treatment: Surgical repair.

#### Chyle fistula

The frequency of chyle fistula is higher in radical resection of the right colon than in that of the left colon and rectum.

Prevention: Dissect the roots of mesenteric vessels using proper electrical devices.

Treatment: Fasting, parenteral nutrition, fat-free diet, delayed removal of abdominal drainage tube.

### Specific complications

Robot-assisted colorectal surgery has some risks related to machine systems, especially for remote surgeries. Accurate control depends on the connecting data quality between the surgeon console and the robot in the operation room. Instruments and electrical equipment are all vulnerable, and the operative robotic system is no exception.

## Postoperative therapy

Closely observe the changes in respiration, body temperature, drainage volume and character, urine volume and color, incision recovery, and so on. Notice whether there is hypercapnia, bleeding in the abdominal cavity, anastomotic bleeding, anastomotic fistula or infection, and so on.

Give proper nutrition support, turn over and pat the back, help with expectoration and reducing phlegm, prophylactically use antibiotics, and exercise urination function early. Early ambulation prevents deep venous thrombosis. As compared with patients who undergo conventional surgery, bowel movements resume significantly earlier in patients who undergo robotic surgery, and their oral intake could be resumed earlier according to their conditions. Patients with stoma should learn related nursing knowledge before discharge.

## Mechanical fault modes and error handling

Error handling is important for the safety of robotic surgery. Faults in the robot system during surgery can generally be categorized as recoverable and non-recoverable fault modes. With a recoverable fault, the indicator lighter on the robotic arm will glow yellow, and the system will trigger an alarm sound. Following instructions on the screen, operation room staff can resolve the fault and continue the procedure. When a non-recoverable fault occurs, the indicator lighter on the robotic arm will glow red, and the system will trigger the alarm. Operation room staff needs to record the error number on the screen (to share it with customer service) and then restart the system. Some non-recoverable faults can be solved this way, and the surgical procedure can go on. However, when a severe fault that cannot be resolved by restarting the system repeatedly occurs, it is necessary to remove the robotic surgical system, convert to laparoscopic or open surgery, and have a maintenance engineer come and repair the system.

There is an emergency brake button on the main console. Do not touch it unless the situation is an emergency.
